# Neural network plasticity in Parkinson's disease: the impact of exercise-driven modulation

**DOI:** 10.3389/fresc.2026.1859762

**Published:** 2026-07-10

**Authors:** M. Alberti, D. Momi, C. Battisti, R. Marconi, F. Ginanneschi, A. Rossi, L. Monti

**Affiliations:** 1 University of Siena, Siena, Italy; 2 Wu Tsai Neurosciences Institute – Stanford University, Stanford, CA, United States; 3 General Hospital “Misericordia”, Grosseto, Italy; 4 Rehabilitative Research Foundation Gianfranco Salvini ETS, Arezzo, Italy; 5 General Hospital “S.Maria alla Gruccia”, Arezzo, Italy

**Keywords:** diffusion imaging, forced exercise, neural network modulation, Parkinson's disease, rehabilitation

## Abstract

**Introduction:**

Parkinson's disease (PD) is characterized by progressive motor impairment and large-scale network dysfunction that extends beyond dopaminergic degeneration. Although rehabilitative exercise improves clinical outcomes, its capacity to induce structural brain plasticity remains incompletely understood. Here, we tested the hypothesis that forced (high-intensity) upper-limb exercise induces white matter microstructural remodeling in PD.

**Material and methods:**

Twenty-three patients with idiopathic PD (mean age 69.1 ± 6.5 years) were allocated to a forced exercise group (FE; *n* = 13) or a voluntary exercise group (VE; *n* = 10) and underwent an 8-week supervised upper-limb training protocol. Groups were matched for age, sex, and clinical severity. Assessment included diffusion tensor imaging (DTI) of cerebellar and thalamocortical tracts, computerized dynamic posturography (CDP), the motor section of the Unified Parkinson's Disease Rating Scale (UPDRS-III) and the Hoehn and Yahr scale.

**Results:**

Significant Group  ×  Time interactions were identified in the inferior cerebellar peduncle mean diffusivity (MD), Axial Diffusivity, Radial Diffusivity (RD); all *p* < 0.001 and middle cerebellar peduncle (MDP) *p* = 0.021), confirming that microstructural changes were significantly larger in the FE group. Results for the anterior corticothalamic radiation represent exploratory trends (*p* ≈ 0.06 for the interaction) and should be interpreted with caution. Findings were accompanied by significant improvements in postural stability in the FE group.

**Conclusion:**

These findings identify exercise-induced plasticity within distributed motor–vestibular networks, supporting a systems-level compensatory mechanism. Our results provide *in vivo* evidence that targeted rehabilitation can reshape structural connectivity in PD, with implications for developing disease-modifying interventions.

## Introduction

Parkinson's disease (PD) is increasingly recognized as a disorder of multiple neural networks, rather than one solely attributable to nigrostriatal dopaminergic degeneration. While dopamine depletion in the basal ganglia remains the defining pathological feature underlying PD's primary motor symptoms, mounting evidence implicates cortico-subcortical and cortico-cerebellar network dysfunction in postural instability, balance impairment, gait disturbances, and tremor (1, 2).

### Cerebellar compensation

The cerebellum plays a key role in motor control, making extensive reciprocal connections with the cerebral cortex, basal ganglia, brainstem and spinal cord (3, 4). It integrates sensory and motor signals to coordinate the timing, precision, and adaptation of movement. In addition to its traditional roles in coordination and motor learning growing evidence suggests that cerebellar circuits play a dynamic role in motor planning and modulation of motor networks, such as partially compensating for basal ganglia dysfunction (4). Cerebellar networks may partially counterbalance basal ganglia dysfunction through increased recruitment of cerebello-thalamo-cortical pathways, particularly during early disease stages (3–6). Functional neuroimaging studies have provided considerable support for this framework, consistently reporting elevated cerebellar activation and enhanced functional connectivity among the cerebellum, thalamus, supplementary motor area (SMA), and primary motor cortex in PD patients (6–9)— changes that appear to sustain motor performance in the face of progressive basal ganglia deterioration. Collectively, these findings have been reinforced by translational work demonstrating that cerebellar output can modulate motor circuits through bidirectional interactions with the basal ganglia, underscoring the existence of an integrated network architecture rather than independent parallel systems (10).

### Diffusion tensor imaging in PD

While functional MRI, PET, and perfusion studies have extensively characterized functional compensatory processes in PD, the microstructural substrates underlying these mechanisms within cerebello-thalamo-cortical pathways remain incompletely understood (11–13). Diffusion MRI (dMRI) offers a powerful framework for investigating microstructural integrity in PD, with existing studies reporting alterations in cerebellar peduncles, thalamocortical projections, and other motor-relevant tracts (6, 11, 14–19) On biological level, reduced radial diffusivity (RD) and mean diffusivity (MD) in cerebellar white matter — with preserved axial diffusivity (AD) — have been linked to demyelination and neuroinflammation, and diffusion tensor imaging (DTI)-derived metrics correlate significantly with disease severity (3, 20). Despite these advances, relatively few studies have specifically examined the integrity of cerebello-thalamo-cortical circuits in relation to postural control, vestibular processing and rehabilitative exercise.

### Exercise paradigms

An equally important gap concerns rehabilitation-induced neuroplasticity. Although exercise is increasingly recognized as a potent modulator of neural plasticity, only a limited number of studies have investigated whether targeted rehabilitation can induce measurable structural remodeling of the white matter pathways involved in compensatory motor control (7, 21–25). Available evidence suggests that forced exercise (FE), in which movement intensity exceeds an individual's preferred voluntary rate, produces greater motor benefits than voluntary exercise. Neuroimaging studies have shown that FE induces changes not only within classical motor cortical regions, such as the supplementary motor area, but also within cerebellar territories, including Crus II. Increased perfusion and activation of these regions following forced exercise suggest the engagement of proprioceptive and sensorimotor integration mechanisms capable of recruiting cerebellar compensatory networks. Given the established role of proprioceptive afferents in modulating cerebellar and vestibular processing, these findings raise the possibility that FE may strengthen communication within cerebello-thalamo-cortical circuits more effectively than voluntary exercise. Recent evidence further supports this interpretation. Supervised upper-limb exercise has been shown to improve vestibular integration and postural stability in PD, whereas voluntary unsupervised exercise fails to produce comparable effects. These findings suggest that the intensity and modality of training are critical determinants of the recruitment of compensatory neural networks. The superior efficacy of forced exercise may therefore reflect its ability to activate residual cerebellar, thalamic, and frontal cortical pathways that remain available for adaptive plasticity despite ongoing neurodegeneration.

### Postural assessment

Impaired vestibular processing has been identified as a key — and independent — determinant of postural instability in PD. Therapeutic strategies targeting vestibular function may represent a valuable approach to improve motor outcomes in PD. Postural stability can be assessed using the Sensory Organization Test (SOT) administered on the EquiTest platform, which measures center-of-gravity sway across six sensory conditions — combining eyes open or closed, sway-referenced surface, and sway-referenced surround — over three 20-second trials per condition. Condition-specific scores quantify the relative contribution of somatosensory, visual, and vestibular inputs to balance control, and are integrated into a composite SOT score reflecting overall postural stability (7, 21, 22, 26).

### Study hypothesis

Upper-limb exercise offers a unique experimental model, as preserved neural coupling between upper and lower limbs may indirectly influence axial and vestibular control (7, 26). Prior work on the same exercise paradigm and patient sample documented significant increases in cerebral blood flow within motor cortex and cerebellar regions selectively in the FE group, while unsupervised voluntary exercise failed to produce comparable neurophysiological benefits (7).Targeted, supervised forced exercise appears to engage non-dopaminergic circuits, enhancing vestibular function and postural control while providing compensatory support for impaired basal ganglia networks (26). Against this background, we hypothesized that a structured forced upper-limb exercise intervention would induce measurable microstructural changes, quantified using DTI metrics (MD, AD, RD), within cerebellar and thalamocortical pathways, and that these changes would be associated with improvements in postural control.

## Material & methods

**Figure 1:**
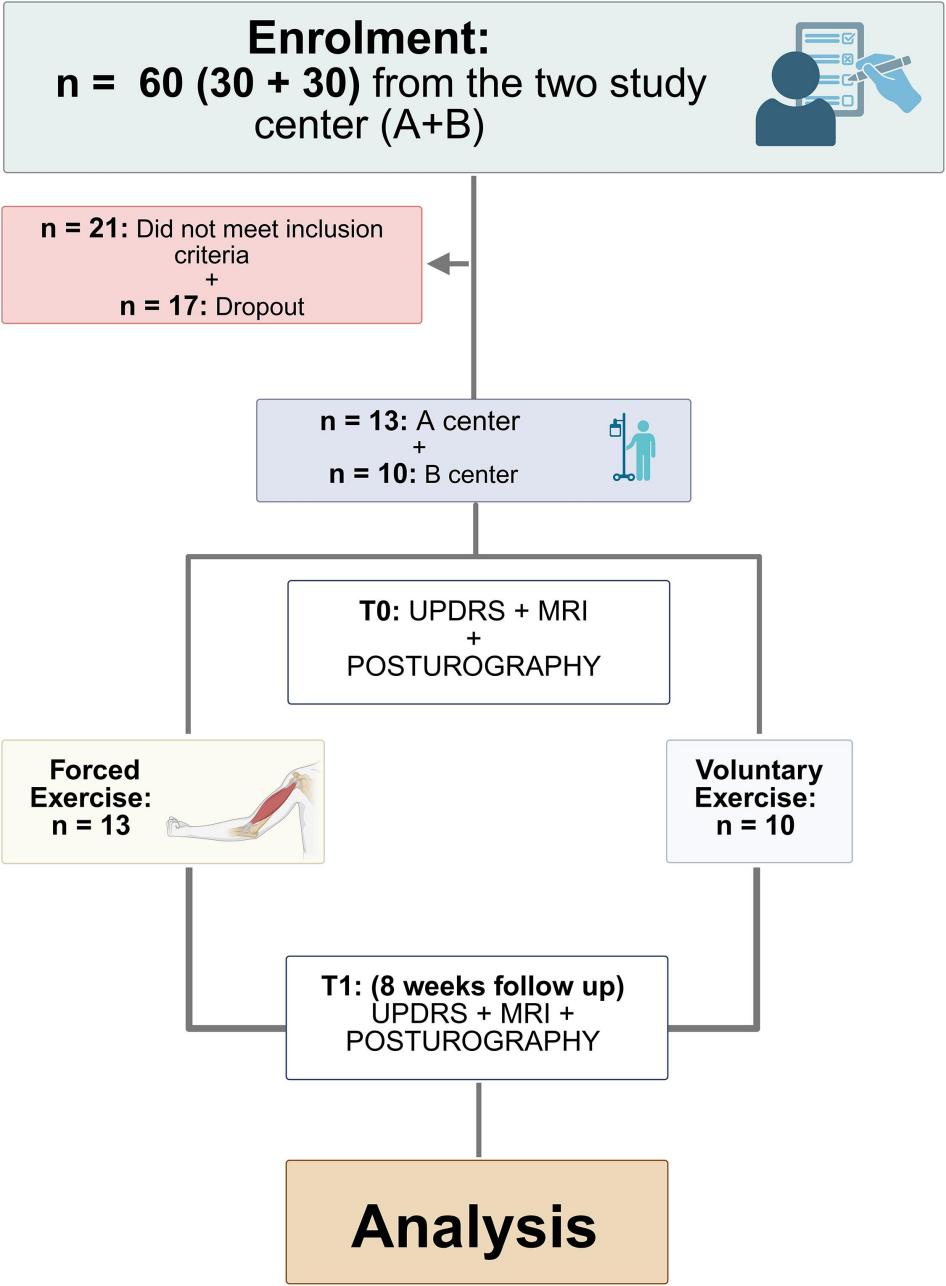
CONSORT-style flow diagram of participant recruitment, allocation, follow-up, and analysis. Sixty patients with Parkinson's disease were initially screened across two study centers (Centers A and B; 30 participants per center). Groups were subsequently matched for age, sex, disease severity, pharmacological therapy, and disease duration to minimize baseline differences. After eligibility assessment, 21 participants did not meet the inclusion criteria and 17 participants discontinued participation before enrollment. A total of 23 participants were included in the study and allocated according to the same rehabilitation protocol available at each center: 13 participants to the Forced Exercise (FE) group (Center A) and 10 participants to the Voluntary Exercise (VE) group (Center B). Baseline and post-intervention evaluations included Unified Parkinson's Disease Rating Scale (UPDRS), magnetic resonance imaging (MRI), and posturographic assessment. After an 8-week follow-up period, no participants were lost to follow-up or discontinued the intervention. All enrolled participants completed the study and were included in the final analysis.

Twenty-three patients with idiopathic Parkinson's disease (PD; mean age 69.1 ± 6.5 years) were enrolled from the Parkinson's outpatient clinics of “Le Scotte” Hospital (Siena) and the Neurology Unit of Grosseto, Italy. The study protocol was approved by the local Ethics Committee (Protocol Code: PARXIFAL_2: 14548; protocol version 1, December 15, 2020) and was conducted in accordance with the Declaration of Helsinki. Following evaluation by a movement disorder specialist, additional eligible patients under external neurological care were also included. Participants were allocated to a forced exercise group (FE; *n* = 13) or a voluntary exercise group (VE; *n* = 10), matched for age, sex, and disease severity. All patients were either on stable dopaminergic therapy for at least two months or, if newly diagnosed, remained drug-naïve throughout the intervention period. All participants provided written informed consent prior to inclusion (Figure 1). Exclusion criteria included moderate-to-severe cardiopulmonary disease, MRI vascular lesion load, uncontrolled hypertension or metabolic disorders, orthopedic or neurological conditions (particularly involving the shoulder or elbow joints), visual impairment, and any contraindication to magnetic resonance imaging (MRI)(Diagram).

### Research design

This was a controlled, parallel-group interventional study. Participants underwent an 8-week supervised upper-limb training program consisting of either externally paced forced exercise (FE) or self-paced voluntary exercise (VE), depending on group allocation. Training was delivered using the Angel's Wings system. Participants completed a 16-session, 8-week upper-limb program using Angel's Wings, a device combining cervical-dorsal spine distension with shoulder rehabilitation via a cable-pulley-weight system (1–7 kg). Each session followed a progressive weight-loading sequence across five 2-minute sets, preceded by a 10-minute warm-up. Subjects were divided into two groups: a Forced Exercise (FE) group, trained at a forced, trainer-guided pace, and a Voluntary Exercise (VE) group, who exercised independently at self-selected intensity. Motor assessment used UPDRS Part-III and Hoehn & Yahr scale at baseline and post-training, administered by a blinded neurologist (27).

### Clinical and instrumental assessments

All participants underwent evaluations at baseline and after the 8-week intervention. The clinical evaluation was performed using the original UPDRS Part-III motor examination and the Hoehn and Yahr scale, at time 0, before the training with Angel's Wings, and at the end of the training period, which was approximately 7 days after the completion of the 8-week exercise protocol. All clinical evaluations were conducted by the same neurologist, who had extensive experience in Parkinson's disease (PD) and was blinded to the participants' group allocation. All assessments were performed in the ON medication state. Secondary assessments included postural control evaluation using a stabilometric platform (NeuroComR International Inc. Instructions for use: EQUITESTR SYSTEMOPERATOR'SMANUAL. Version 8. Clackamas: NeuroComR International Inc. (2003). All subjects underwent brain MRI and functional connectivity assessments. Post-intervention MRI acquisitions were performed within one week following the completion of the training protocol in both the forced and voluntary exercise groups.

### dMRI: acquisition protocol & preprocessing

Diffusion MRI data were acquired on a Siemens 1.5 T Avanto scanner using a 2D EPI diffusion sequence (ep2d_diff_tensor_64_gap0; TE = 81 ms, TR = 7,100 ms). The protocol comprised 67 volumes: 64 non-overlapping whole-sphere diffusion-encoding directions acquired at a single b-value shell (b = 1,000 s/mm²) and 3 non-diffusion-weighted volumes (b = 0 s/mm²). Preprocessing was performed using a custom pipeline combining FSL and MRtrix3, and included the following steps applied in sequence: thermal noise removal using the Marchenko-Pastur PCA algorithm (MP-PCA; MRtrix3) (28, 29), Gibbs ringing suppression (MRtrix3) (29), eddy-current and subject motion correction (FSL eddy) (30), and bias-field correction (ANTs) (31).

### Tractography & ROI selection

**Figure 2:**
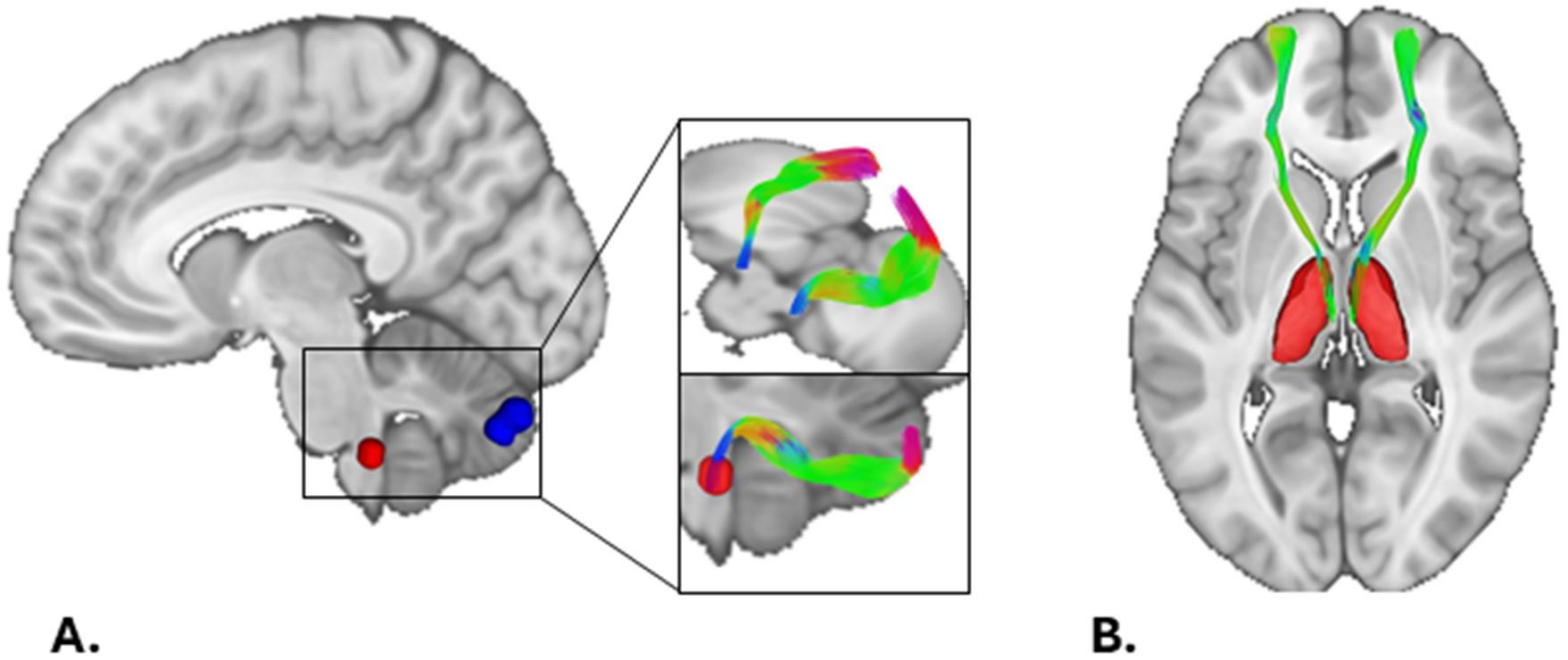
White matter tracts of the inferior cerebellar peduncle and anterior corticothalamic radiation. **(A)** Sagittal view of the seed regions of interest (ROIs) used for tractography of the ICP and example ICP tractography. Seed ROIs are shown in red (starting point), while target ROIs are shown in blue (end point). **(B)** Axial view of the bilateral reconstruction of the anterior corticothalamic tract. All images are overlaid on the MNI152 standard brain. Tract reconstructions were obtained using deterministic tractography and are shown for illustrative purposes to confirm anatomical plausibility of the approach. Seed starting ROIs in red. White matter tracts are displayed using a RGB color code representing the principal spatial orientation of the white matter fiber tracts. Standardized convention maps the 3D space as follows: Red: Left-Right (*X*-axis), Green: Anterior-Posterior (*Y*-axis) and Blue: Superior-Inferior/Dorsal-Ventral (Z-axis).

Whole-brain deterministic streamline tractography was performed in DSI Studio (32) using a combined atlas-based and ROI-to-ROI approach. Regions of interest (ROIs) were defined to constrain reconstruction to anatomically plausible pathways, ensuring that only target tracts were resolved. ROIs were first defined in MNI standard space and subsequently warped to individual dMRI space via nonlinear coregistration (ANTs) (31), followed by thresholding to minimize partial-volume contamination. Diffusion tensors were fitted voxelwise using FSL dtifit, yielding following scalar maps: MD, AD, and RD (30). Tract-averaged diffusion metrics were then extracted by applying the reconstructed streamline masks to each scalar map, enabling quantitative characterization of white matter microstructure within each pathway of interest (Figure 2).

### Statistical analysis

Diffusion metrics were screened for outliers (±2 SD from the mean) prior to analysis. For tracts with bilateral representation (inferior cerebellar peduncle and anterior corticothalamic radiation), diffusivity values from the left and right hemispheres were modeled jointly using linear mixed-effects models (LME) with a random intercept per subject, implemented in R (lme4 package) (33). Denominator degrees of freedom were estimated using the Satterthwaite approximation (lmerTest package) (33).

Time (T0/T1) and Group (Forced vs. Voluntary) were entered as fixed effects, with their interaction (Group  ×  Time) constituting the primary inferential test (34). This approach accounts for the non-independence of bilateral hemisphere measurements within the same individual and directly addresses whether the magnitude of change over time differed between groups, avoiding the statistical fallacy of comparing within-group *p*-values across groups. All *p*-values were corrected for multiple comparisons using the Benjamini–Hochberg false discovery rate (FDR) procedure (35). Findings surviving FDR correction are interpreted as confirmatory; uncorrected marginal results are described as exploratory. Secondary within-group descriptive comparisons (Wilcoxon signed-rank tests) are reported for completeness but were not used for inferential conclusions. Where data were not normally distributed (Shapiro–Wilk *p* < 0.001), non-parametric equivalents were used for descriptive verification. The longitudinal within-subject design of the present study further strengthens our design, as repeated measures on the same individuals substantially reduce the influence of the high interindividual variability that characterizes dMRI. The analysis focused specifically on the inferior and middle cerebellar peduncles, as well as the anterior corticothalamic radiation (35). These tracts were selected using a data-driven strategy that integrated structural imaging findings with functional and posturographic measures. (NeuroComR International Inc. Instructions for use: EQUITESTRSYSTEMOPERATOR'SMANUAL. Version 8. Clackamas: NeuroComR International Inc. (2003).

## Results

Training resulted in region-specific microstructural changes in white matter pathways (Table 1).

**Figure 3:**
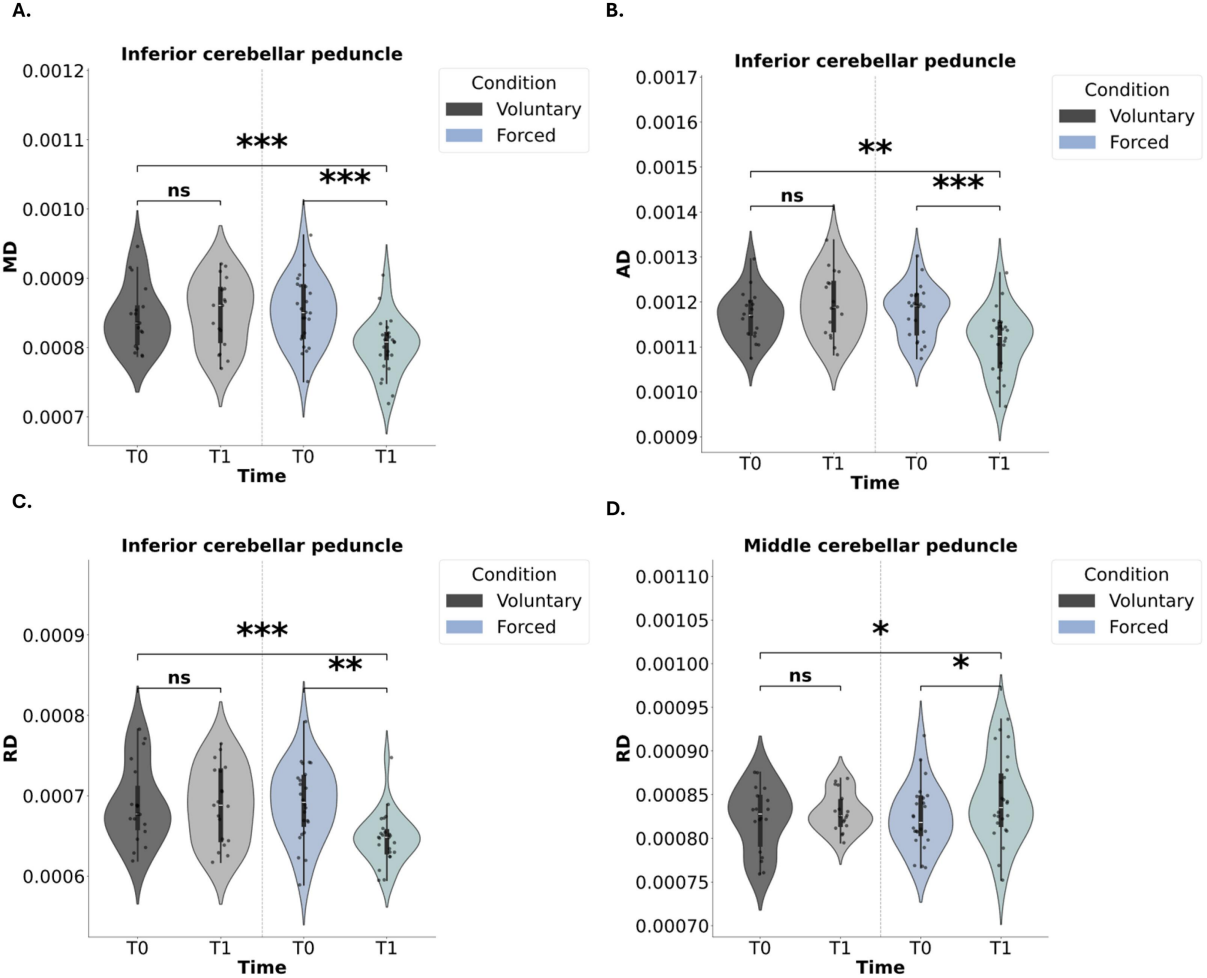
DTI diffusivity metrics in the inferior cerebellar peduncle (ICP) and middle cerebellar peduncle (MCP) following upper-limb exercise training. Mean diffusivity [MD; **(A)**], axial diffusivity [AD; **(B)**], and radial diffusivity [RD; **(C)**] in the ICP, and MD in the MCP **(D)**, are shown for the forced exercise (FE) and voluntary exercise (VE) groups at baseline (T0) and after 8 weeks of training (T1). In the ICP, a significant Group  ×  Time interaction was observed for all three diffusivity metrics [MD: *F*(1,20) = 28.4, *p* < 0.001; AD: *F*(1,20) = 22.1, *p* < 0.001; RD: *F*(1,20) = 19.7, *p* < 0.001; all FDR-corrected], with within-group comparisons confirming significant reductions exclusively in the FE group (all *p* < 0.001), consistent with improved axonal coherence and adaptive myelin remodeling, and no significant changes in the VE group (all *p* > 0.15). In the MCP, a significant Group  ×  Time interaction was similarly observed for MD [*F*(1,20) = 6.3, *p* = 0.021, FDR-corrected], *post-hoc* t-test, FE p. < 0.05; with a selective reduction in the FE group after training, consistent with enhanced fiber coherence or myelin integrity within this pontocerebellar tract, and no corresponding change in the VE group. Error bars represent standard error of the mean.

**Figure 4:**
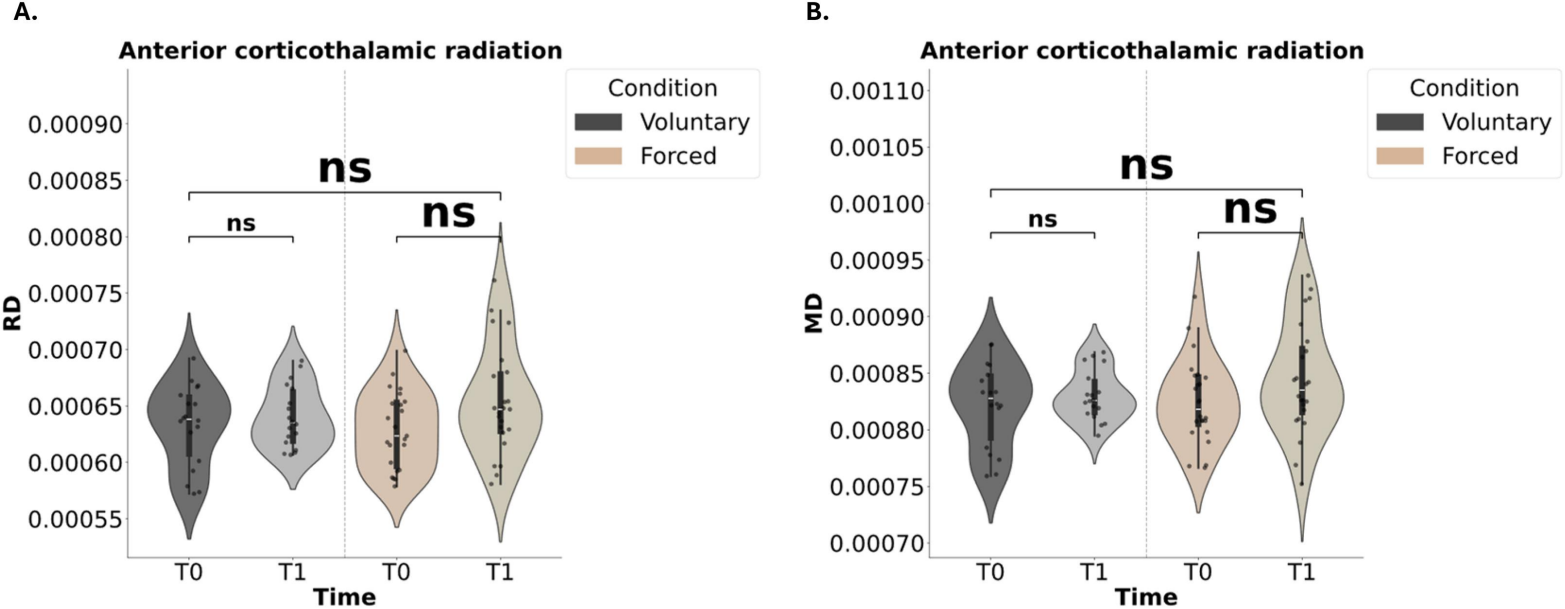
DTI diffusivity metrics in secondary white matter pathways following forced upper-limb exercise. **(A,B)** and **(C)** Mean diffusivity (MD) and Radial diffusivity (RD) in the anterior corticothalamic radiation (ACR): lateralized reductions were observed as exploratory trends in the FE group, the interaction effect did not reach significance after correction (*p* ≈ 0.06), and between-group delta comparisons did not reach statistical significance for these tracts. No significant changes were observed in the voluntary exercise (VE) group for either measure.

**Figure 5:**
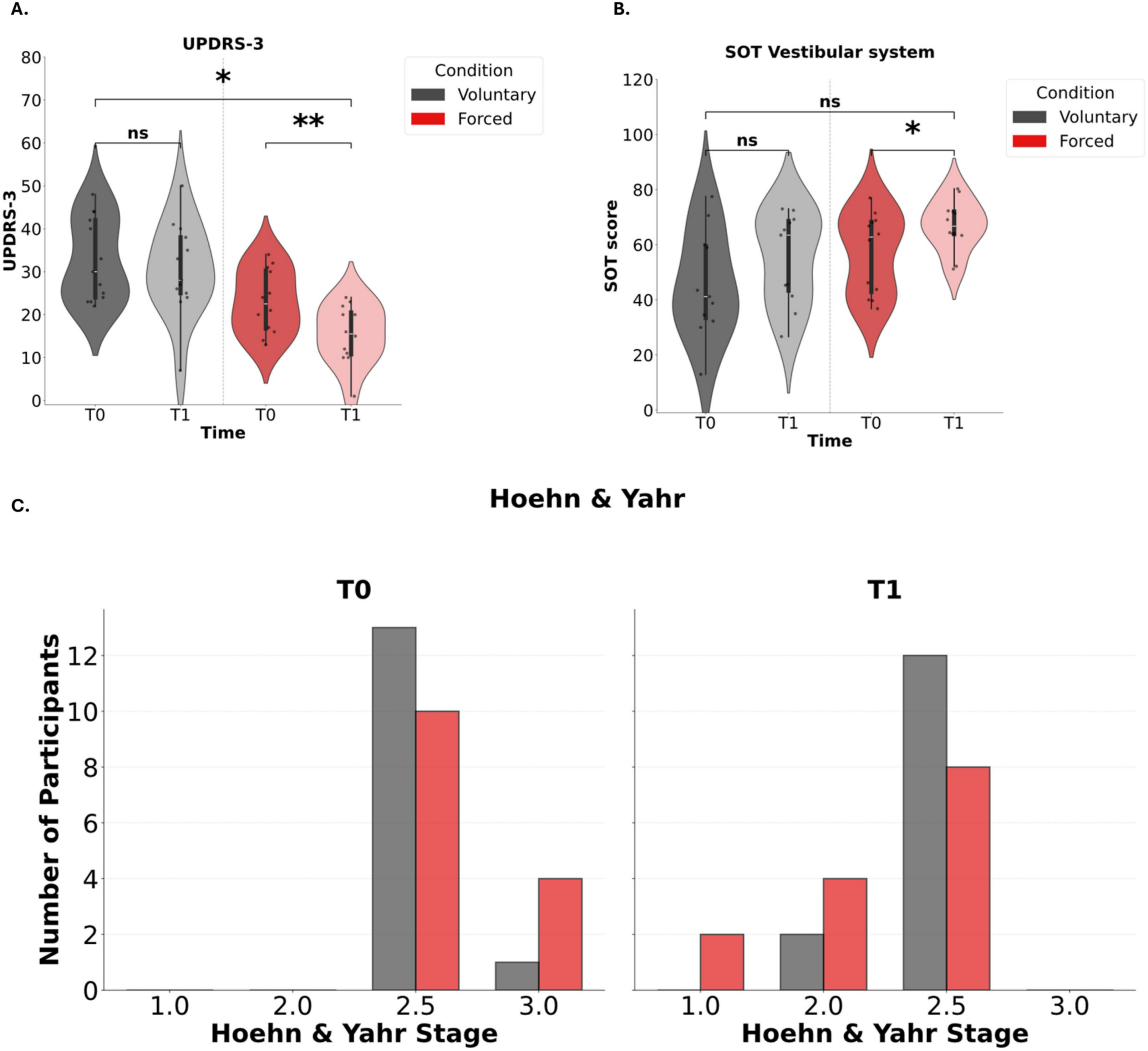
Clinical and vestibular outcomes before (T0) and after (T1) 8 weeks of exercise training in voluntary (VE, grey) and forced exercise (FE, red) groups. **(A)** UPDRS-3 motor scores showed a significant main effect of condition [F(1,23) = 1.23, *p* = 0.001106] and a significant interaction effect [F(1,23) = 1.23, *p* = 0.030353]. Group pairwise t-test: VE *t* = 1.56, *p* = 0.144, FE *t* = 3.80, *p* = 0.0029. **(B)** Sensory Organization Test (SOT) vestibular condition (SOT5) scores. SOT showed a significant main effect of condition [mixed ANOVA, F(1,21) = 1.21, *p* = 0.028772], with no interaction effect. Group pairwise t-test: VE *t* = −1.34, *p* = 0.209 & FE *t* = −2.71, *p* = 0.017. **(C)** Distribution of Hoehn & Yahr stages at baseline and post-training for both exercise conditions (mean values: FE-T0: 2,64 +/- 0.23; FE-T1: 2,14 +/- 0.53 & VE-T0: 2.54 +/- 0.13; VE-T1: 2.43 +/- 0.18). Together, these findings suggest that forced exercise preferentially improves motor performance and vestibular integration compared with voluntary exercise in Parkinson's disease.

**Inferior Cerebellar Peduncle (ICP):** The 2 × 2 Group  ×  Time LME interaction was significant for all three diffusivity metrics [MD: F(1,20) = 28.4, *p* < 0.001; AD: F(1,20) = 22.1, *p* < 0.001; RD: F(1,20) = 19.7, *p* < 0.001; all FDR-corrected], confirming that microstructural changes from T0 to T1 were significantly greater in the FE group. As secondary descriptive information (not used for inferential conclusions), within-group Wilcoxon tests confirmed reductions in MD, AD, and RD in the FE group (all *p* < 0.001), with no corresponding changes in the VE group (all *p* > 0.15) (Figures 3A–C).
**Middle Cerebellar Peduncle (MCP):** The Group  ×  Time interaction was significant [F(1,20) = 6.3, *p* = 0.021], indicating a selective reduction in MD in the FE group and no change in the VE group (Figure 3D).
**Anterior Corticothalamic Radiation (ACR):** The Group  ×  Time interaction for MD and RD showed a statistical trend that did not reach significance after FDR correction [MD: F(1,20) = 4.1, *p* = 0.057; RD: F(1,20) = 3.8, *p* = 0.066] (Figure 4). These results are therefore classified as exploratory and should be interpreted with caution. Between-group delta comparisons did not reach statistical significance for any of these secondary tracts, consistent with the interaction analysis. Overall, the pattern of diffusivity changes is consistent with improved microstructural organization, potentially reflecting enhanced axonal coherence and/or adaptive myelin remodeling induced by training.
**Clinical and postural assessment (**Figure 5**):** Between conditions and within participants Mixed ANOVA on UPDRS-III scores revealed a significant main effect of condition [F(1,23) = 1.23, *p* = 0.001] and a significant time  ×  group interaction [F(1,23) = 1.23, *p* = 0.030] (Figure 5A). Pairwise comparisons confirmed that UPDRS-III scores improved significantly in the FE group (t = 3.80, *p* = 0.003) but not in the VE group (t = 1.56, *p* = 0.144), suggesting that forced exercise selectively drives motor recovery (Figure 5A). Vestibular SOT showed a significant main effect of condition [F(1,21) = 1.21, *p* = 0.029], with no significant interaction effect. Group-level pairwise comparisons indicated a significant improvement in SOT scores following FE (t = −2.71, *p* = 0.017), whereas VE did not produce a significant change (t = −1.34, *p* = 0.209) (Figure 5B). Hoehn & Yahr scores: FE group (mean ± standard deviation) T0: 2,64 ± 0.23, T1: 2,14 ± 0.53; VE (mean ± standard deviation) T0: 2.54 ± 0.13, T1: 2.43 ± 0.18) – Mixed Anova didn't display any statistical difference (Figure 5C).

**Table 1:** Between-group comparison of clinical and diffusion tensor imaging outcomes in FE (forced) and VE (voluntary) groups.

Metric	Anatomical/Functional node	FE Group	VE Group	*P*-value
DTI – MD	Inferior Cerebellar Peduncle	↓ Significant	No Change	*p* < 0.001
DTI – AD	Inferior Cerebellar Peduncle	↓ Significant	No Change	*p* < 0.001
DTI – RD	Inferior Cerebellar Peduncle	↓ Significant	No Change	*p* < 0.001
DTI – MD	Middle Cerebellar Peduncle	↓ Significant	No Change	*p* = 0.021
DTI – MD	Ant. Corticothal. Rad.	↓ Trend (exploratory)	No Change	*p* = 0.057
DTI – RD	Ant. Corticothal. Rad.	↓ Trend (exploratory)	No Change	*p* = 0.066
CDP – SOT	Equilibrium Score (ES)	↑ Significant	Minimal Change	*p* > 0.05
UPDRS-III	Total Motor Score	↓ Impairment	Minimal Change	*p* = 0.030
Therapeutical Dose	Levodopa	672,1 mg	582,2 mg	ns (*p* = 0,48)
Disease duration	//	6,9 Yrs	7Yrs	ns

Disease duration was comparable between groups (FE: 6.9 years; VE: 7.0 years; n.s.). A significant improvement in postural control was observed in the FE group as assessed by CDP–SOT Equilibrium Score (ES), whereas no change was detected in the VE group (*p* = 0.017).For motor severity (UPDRS-III), the FE group demonstrated a significant improvement following treatment (pre-treatment: 25.00 ± 9.36; post-treatment: 19.23 ± 11.62; t = 2.77; *p* ≈ 0.017), while the VE group showed no significant change (pre-treatment: 29.00 ± 11.65; post-treatment: 28.40 ± 8.72; t = 0.13; *p* ≈ 0.90).Therapeutic dose did not differ significantly between groups (FE: 672.1 mg; VE: 582.2 mg; *p* = 0.48).DTI analysis revealed a significant reduction in mean diffusivity (MD) in the inferior cerebellar peduncle (*p* < 0.01) and anterior corticothalamic radiation (*p* = 0.045) in the FE group, whereas no significant changes were observed in the VE group.

## Discussion

The present study provides converging clinical and neuroimaging evidence that forced upper-limb exercise induces measurable microstructural plasticity in PD, with specific involvement of cerebellar and thalamocortical pathways. These findings extend the current understanding of exercise in PD beyond symptomatic improvement, demonstrating that targeted rehabilitation can reshape structural connectivity within distributed motor networks. Changes in MD, AD, and RD, particularly decreases, are commonly interpreted as reflecting alterations in axonal organization, myelin integrity, and neuroinflammation (14), underscoring the release of BDNF or astrocyte swelling (36, 37). This suggests that the observed effects may represent activity-dependent mechanisms within cerebellar circuitry.

### Cerebellar compensation and basal ganglia offset

Within the context of PD, these cerebellar changes are particularly relevant, as the cerebellum is increasingly recognized as a compensatory hub capable of partially offsetting basal ganglia dysfunction. This interpretation is further supported by recent connectome evidence demonstrating that deep brain stimulation of the GPi produces differential axial gait outcomes depending on cerebellar subregion connectivity, with connectivity to sensorimotor lobules (I–V) associated with gait improvement and connectivity to cerebellar Crus II associated with worsening axial symptoms (4, 9). The convergence of exercise-induced white matter plasticity observed in the present study with stimulation-based connectivity mapping identifies the inferior cerebellar peduncle and sensorimotor cerebellar territory as key nodes for favorable axial outcomes, reinforcing the broader thesis that cerebello-thalamo-cortical networks represent tractable therapeutic targets for postural instability and gait dysfunction in PD.

### Postural stability and cerebellar-vestibular circuits

The involvement of cerebellar pathways provides a mechanistic bridge to the observed improvements in postural stability. Postural control critically depends on the integration of vestibular, visual, and somatosensory signals, with the cerebellum playing a central role in sensory reweighting (1, 8). In PD, impaired central vestibular processing has been documented despite preserved peripheral function, leading to deficits in balance and increased fall risk (1). The present findings suggest that rehabilitative exercise, particularly when delivered in a forced, high-intensity paradigm, can enhance the efficiency of these cerebellar–vestibular circuits. This interpretation is further supported by neurophysiological evidence indicating that activation of cervical and shoulder girdle musculature can modulate vestibular gain via cerebellar integration pathways. In parallel, the observed trends in the anterior corticothalamic radiation point to potential plasticity within thalamocortical circuits, which are essential for motor planning, execution, and the integration of sensory feedback.

### Statistical approach and group comparisons

Group-level conclusions are based on the 2 × 2 Group  ×  Time interaction test rather than solely on a comparison of within-group *p*-values, the latter being an inferential approach known to be insufficient. The significant interactions observed for the ICP (all metrics, *p* < 0.001) and MCP (*p* = 0.021) provide formal statistical evidence that these changes were selectively driven by the forced exercise paradigm. For the ACR, the interaction did not reach significance after correction (*p* ≈ 0.06); these results are therefore described as exploratory trends, and between-group delta comparisons did not reach statistical significance for these tracts. This is consistent with the interpretation that the ACR findings are preliminary and warrant replication (38–40) in larger samples.

The present findings are consistent with previous functional neuroimaging studies demonstrating that forced exercise enhances motor cortex activation and functional connectivity patterns, further corroborating the hypothesis that exercise intensity is a critical determinant of neuroplastic outcomes in PD (6, 9, 21–23, 25–27).

### Limitations

The relatively small sample size (FE: *n* = 13; VE: *n* = 10) limits statistical power and generalizability. The bilateral tract analysis, which treats left and right hemisphere values from the same subject as paired observations within a mixed-effects framework, appropriately accounts for within-subject correlation but also reduces the effective degrees of freedom relative to a purely subject-level analysis; replication in larger cohorts is therefore essential. Additionally, results for the anterior corticothalamic radiation did not survive FDR correction for the Group  ×  Time interaction and should be considered exploratory. While DTI provides indirect measures of microstructure, it cannot fully disentangle the specific biological substrates underlying diffusivity changes. The absence of a healthy control group precludes determination of whether the observed DTI changes represent disease-specific plasticity or reflect more general training-related effects common to aging populations. The study also lacks long-term follow-up data, so the durability of the microstructural changes and their functional correlates remain unknown. Furthermore, the potential confounding effects of concurrent pharmacotherapy (e.g., levodopa dose adjustments during the intervention period) were not systematically controlled, and their influence on DTI metrics cannot be excluded, as well as biased tract selection and individual differences in tract sizes. Finally, the pre–post design does not permit causal inference: while the observed DTI changes co-occur with clinical improvement in the FE group, a direct causal relationship between structural plasticity and functional benefit cannot be established based on the present data alone. Future studies combining diffusion imaging with myelin-sensitive imaging or multimodal approaches (e.g., DTI and functional MRI) will be essential to further elucidate the mechanisms of exercise-induced plasticity.

## Conclusions

Forced upper-limb exercise appears to be a key driver of network-level white matter plasticity in Parkinson's disease, particularly within cerebellar and thalamocortical pathways. These findings suggest that structured, intensity-driven exercise is associated with measurable microstructural remodeling of white matter, providing preliminary mechanistic evidence linking rehabilitation to structural brain changes. This supports the development of targeted, high-intensity therapeutic strategies to harness residual neuroplasticity and improve motor and vestibular function in PD, while acknowledging that replication in larger controlled studies is needed.

## Data Availability

The raw data supporting the conclusions of this article will be made available by the authors, without undue reservation.
